# Antibody-Dependent NK Cell Activation Is Associated with Late Kidney Allograft Dysfunction and the Complement-Independent Alloreactive Potential of Donor-Specific Antibodies

**DOI:** 10.3389/fimmu.2016.00288

**Published:** 2016-08-11

**Authors:** Tristan Legris, Christophe Picard, Dilyana Todorova, Luc Lyonnet, Cathy Laporte, Chloé Dumoulin, Corinne Nicolino-Brunet, Laurent Daniel, Anderson Loundou, Sophie Morange, Stanislas Bataille, Henri Vacher-Coponat, Valérie Moal, Yvon Berland, Francoise Dignat-George, Stéphane Burtey, Pascale Paul

**Affiliations:** ^1^Nephrology Dialysis Renal Transplantation Center, Assistance Publique des Hôpitaux de Marseille, Hospital de la Conception, Marseille, France; ^2^Établissement Français du Sang Alpes Méditerranée, Marseille, France; ^3^ADES UMR 7268, CNRS, EFS, Aix-Marseille Université, Marseille, France; ^4^UMR 1076, Vascular Research Center of Marseille, INSERM, Aix-Marseille University, Marseille, France; ^5^Hematology Unit, Assistance Publique des Hôpitaux de Marseille, Hopital de la Conception, Marseille, France; ^6^Laboratory for Anatomy, Pathology, Neuropathology, Hôpital de la Timone, Aix-Marseille University, Marseille, France; ^7^Unité d’Aide méthodologique à la Recherche Clinique et Epidémiologique, DRRC, Assistance Publique Hôpitaux de Marseille, Marseille, France; ^8^Centre d’Investigation Clinique, Hôpital de la Conception, Marseille, France

**Keywords:** natural killer cells, kidney transplantation, donor-specific antibodies, antibody-dependent cellular cytotoxicity, antibody-mediated rejection

## Abstract

Although kidney transplantation remains the best treatment for end-stage renal failure, it is limited by chronic humoral aggression of the graft vasculature by donor-specific antibodies (DSAs). The complement-independent mechanisms that lead to the antibody-mediated rejection (ABMR) of kidney allografts remain poorly understood. Increasing lines of evidence have revealed the relevance of natural killer (NK) cells as innate immune effectors of antibody-dependent cellular cytotoxicity (ADCC), but few studies have investigated their alloreactive potential in the context of solid organ transplantation. Our study aimed to investigate the potential contribution of the antibody-dependent alloreactive function of NK cells to kidney graft dysfunction. We first conducted an observational study to investigate whether the cytotoxic function of NK cells is associated with chronic allograft dysfunction. The NK-Cellular Humoral Activation Test (NK-CHAT) was designed to evaluate the recipient and antibody-dependent reactivity of NK cells against allogeneic target cells. The release of CD107a/Lamp1^+^ cytotoxic granules, resulting from the recognition of rituximab-coated B cells by NK cells, was analyzed in 148 kidney transplant recipients (KTRs, mean graft duration: 6.2 years). Enhanced ADCC responsiveness was associated with reduced graft function and identified as an independent risk factor predicting a decline in the estimated glomerular filtration rate over a 1-year period (hazard ratio: 2.83). In a second approach, we used the NK-CHAT to reveal the cytotoxic potential of circulating alloantibodies *in vitro*. The level of CD16 engagement resulting from the *in vitro* recognition of serum-coated allogeneic B cells or splenic cells was further identified as a specific marker of DSA-induced ADCC. The NK-CHAT scoring of sera obtained from 40 patients at the time of transplant biopsy was associated with ABMR diagnosis. Our findings indicate that despite the administration of immunosuppressive treatments, robust ADCC responsiveness can be maintained in some KTRs. Because it evaluates both the Fab recognition of alloantigens and Fc-driven NK cell activation, the NK-CHAT represents a potentially valuable tool for the non-invasive and individualized evaluation of humoral risk during transplantation.

## Introduction

Kidney transplantation is the best treatment for patients with end-stage chronic kidney disease (CKD). However, long-term graft survival is limited by numerous factors, including inadequate control of the immune response to the allograft vasculature (1–3). Antibody-mediated rejection (ABMR) is a major cause of long-term transplant failure (2, 4, 5). *De novo* donor-specific antibodies (*dn*DSA) have been identified as major contributors to chronic ABMR and have been associated with graft microvascular injury (6–9) and arterial intimal fibrosis (10, 11). Despite advances in the development of the multiplex solid-phase single-antigen bead assay (SABA), which detects circulating donor-specific antibodies (DSAs) (12, 13) and their capacity to bind C1q or C3d (14–18), predicting the graft outcome in sensitized transplant recipients remains difficult (16, 19–23). A better understanding of the underlying mechanisms that contribute to ABMR is thus of key importance for improving therapeutic strategies. Recent findings indicate that, in addition to complement-dependent mechanisms, antibody-dependent cellular cytotoxicity (ADCC) involving γδ T cells or natural killer (NK) cells contributes to DSA-mediated graft injury (24–33). The NK cell molecular signatures from transplant biopsies of patients with ABMR (32, 34–37) suggest that NK cell activation is associated with humoral allograft vasculopathy (38, 39). Although NK cells are known cytotoxic effectors of the innate immune responses to antibodies, their potential pathogenic role in transplant rejection remains poorly documented. The antitumor efficiency of therapeutic monoclonal antibodies has been shown to be dependent on the expression of CD16-FcRγIIIA on NK cells (40, 41). FcγR polymorphisms have also been suggested as predisposing factors to infectious complications after liver transplantation (42). Similarly, we hypothesized that antibody-dependent cytotoxicity may be partly conditioned by the intrinsic capacity of the recipient NK cells to form conjugates with antibody-coated donor cells. In the transplant setting, the recipient NK cell alloreactivity may be affected by numerous factors, such as immunosuppressive drugs, infection, uremia, and inflammatory stresses (27, 43–48). Therefore, we aimed to investigate the potential link between NK cell cytotoxicity and allograft function in a cohort of late kidney transplant recipients (KTRs). An *in vitro* NK-Cellular Humoral Activation Test (NK-CHAT) was designed to address the following: (1) the potential link between NK cell activation and transplant dysfunction and (2) the potential toxicity of *dn*DSAs in promoting NK cell activation.

## Materials and Methods

### Patients and Study Design

Study approval was obtained from the Agence Française de Sécurité Sanitaire (Afssaps Ref B805-1860) and from the Comité de Protection des Personnes SUD Méditerranée I. The study was supervised by the Institut National de la Santé et de la Recherche Médicale (INSERM, protocol granted in 2008 under Ref ID RCB 2008-A00604-51, C07-17).

Kidney transplant recipients were prospectively enrolled in the study from November 2008 to November 2011 at the Centre de Nephrologie et Transplantation Rénale, Hôpital de la Conception, Marseille. Patients considered for inclusion underwent transplantation between 2001 and 2005 (>3 years post graft) as part of a follow-up for a previous study (49). Patients with insufficient peripheral blood mononuclear cells (PBMCs) to perform the functional test or patients lacking follow-up data for kidney graft function after 1 year were not considered in the analysis (*n* = 42). The distribution of the variables for this group of 42 patients with missing data were not significantly different from that found for the cohort of 148 KTRs who were included in the final study cohort. The baseline characteristics of the 148 KTRs are summarized in Table 1. Renal function decline during follow-up was defined as either a 10% loss in the estimated glomerular filtration rate (eGFR) (mL/min/1.73 m^2^, defined with the MDRD simplified equation, *n* = 51) or renal graft failure (*n* = 9). The control group was composed of 86 volunteer blood donors without renal failure.

**Table 1:** **Characteristics of the 148 KTRs according to transplant function**.

Variable	KTR with eGFR < 60	KTR with eGFR ≥ 60	*p* value	CTL
			KTR with eGFR < 60 versus KTR with eGFR ≥ 60	
**Recipient *n*** = 148	92 (62%)	56 (38%)		86
Men (%)	55 (60%)	36 (64%)	ns	47 (55%)
Age at transplant (year, mean ± SD)	45 ± 13	32 ± 13	0.07	NA
Body mass index (kg/m^2^) at transplant	23 ± 4**	23 ± 4*	ns	25 ± 5
**Donor**
Men (%)	63 (67.7%)	41 (74.5%)	ns	N.A
Age (year, mean ± SD)	45 ± 13	32 ± 13	<0.0001	N.A
No of HLA mismatch (min–max)	3 (0–5)	3 (1–4)	ns	N.A
**Antecedent/risk factors**				
Time on dialysis, months	33 (18.5–55.5)	25 (14–52)	ns	N.A
Second or third transplantation	12 (13%)	6 (11%)	ns	N.A
Delayed graft function	24 (26%)	8 (14.5%)	0.091^t^	N.A
eGFR at 1 year posttransplant (M12)	45 ± 13	63 ± 11.5	<0.0001	N.A
Acute rejection	8 (8.7%)	1 (1.8%)	0.088	N.A
**Characteristics at time of inclusion**
Time post graft (year, mean ± SD)	6.3 ± 1.8	6.03 ± 1.7	ns	N.A
Serum creatinine, μmol/L (mean ± SD)	161 ± 58***	89 ± 14***	<0.0001	73 ± 14
eGFR (mL/min/1.73 m^2^, mean ± SD)	41.3 ± 11***	75.6 ± 13***	<0.0001	96 ± 18
Obesity (BMI > 30)	13 (14.1%)	4 (7.1%)	0.196	N.A
Current smokers	17 (18.5%)	4 (7.1%)	0.055	N.A
Diabetes	18 (19.6%)	8 (14.3%)	ns	N.A
Hypertension	91 (99%)	51 (91.1%)	0.019	N.A
Cardiovascular history	16 (17.4%)	17 (30.4%)	ns	N.A
DSA at time of inclusion	13 (14%)	2 (4%)	0.039	N.A
**Immunosuppressive therapy**
Cyclosporine	42 (46%)	21 (37.5%)	ns	N.A
Mycophenolate mofetil	46 (49.5%)	32 (58.2%)	ns	N.A
Azathioprine	22 (24%)	16 (29%)	ns	N.A
Steroids	86 (92.5%)	51 (92.8%)	ns	N.A
**NK cell number and cytotoxic function**
% NK cells (median, 25–75 pct)	9.1 (5.6–15)	10.1 (6.5–15)	ns	10.4 (9–14.5)
Number of NK cells/mm^3^ (median, 25–75 pct)	118 (88–180)	149 (93–224)	0.103^t^	197 (142–264)
**NK cell activation toward target (%CD107a/Lamp1)**
Natural cytotoxicity toward B cells	5.8 (4.3–9.1)	5.4 (3.7–8.6)	ns	6.9 (4.3–11)
ADCC: B cells + rituximab	29.4 (18.7–37)	22.6 (15–39)	0.008	31 (25–40)
**eGFR loss** ≥10%	33 (36%)	18 (32%)	ns	N.A

The *p* values from the comparison of KTRs and healthy individuals (eGFR ≥ 60, CTL) were used to assess the significance of the differences (*p < 0.05, **p < 0.01, ***p < 0.0001); ^t^shows the differences with *p* values <0.2, and ns indicates the non-significant differences (*p* > 0.2). The progression of CKD was evaluated as an eGFR decrease of at least 10% from the time of inclusion in the study (time of NK cell evaluation) to 1 year later. According to the distribution of variables, the results are shown as numbers and percentages, means ± SD, or medians (25–75th percentile ranges).

### Cells, Plasma, and Sera

Peripheral blood mononuclear cells for use as effector cells were obtained from healthy volunteer blood donors and KTR patients and were isolated using a Ficoll gradient. The cells used as targets in the NK-CHAT assay included the following: cells from the K562 cell line, cells from a B-EBV immortalized cell line [human leukocyte antigen (HLA)-genotype Table 2], NK cell-depleted PBMCs, and the residual donor spleen cells from the pretransplant cross-matching. The splenic or renal tissues were minced and homogenized by incubation with 50 μg/mL collagenase 1A (Sigma C9891) for 30 min at 37°C. Plasmapheresis samples (500 mL), containing HLA-A2 or HLA-DR4 DSA, were collected during the first round of ABMR desensitization therapy from two patients. KTR serum samples were obtained before the initiation of ABMR treatment and blindly analyzed for NK-CHAT activity. NK-CHAT standardization was achieved using a monoclonal therapeutic IgG-recognizing CD20, rituximab (obtained from the residual samples that could not be used in the clinics or that were beyond the expiration date). The samples were obtained under institutional approval provided by the Pharmacy and Nephrology Department of Assistance Publique des Hôpitaux de Marseille.

**Table 2:** **Univariate and multivariate analysis of factors associated with the progression to CKD at 1 year post-enrollment into the study**.

≥10% eGFR loss	Univariate analysis	Estimates: asymptotic Cox regression multivariate					
Variables in the model	*p*	*B*	SE	*p*	HR	95% CI for Exp (*B*) (lower–upper)	
CD107a/Lamp1URI >3	0.033	1.04	0.50	0.038	2.83	1.06	7.57
eGFR 1 year after transplantation	0.001	−0.06	0.02	0.001	0.94	0.91	0.98
eGFR at time of evaluation	0.386	0.03	0.01	0.036	1.03	1.00	1.05
Maintenance treatment: azathioprine	0.044	0.93	0.48	0.052	2.53	0.99	6.47
Smoking of time of enrollment	0.179^t^	−0.05	0.44	0.273	0.62	0.26	1.46
Presence of DSA at time of enrollment	0.214	−0.55	0.65	0.401	0.58	0.16	2.07
Recipient age at transplant ≥55 years	0.157^t^	−0.32	0.39	0.416	0.73	0.34	1.57
Time posttransplant at time of enrollment	0.333	0.05	0.11	0.640	1.05	0.85	1.30
Number of NK cells at time of enrollment	0.192^t^	−0.001	0.002	0.688	1.00	1.00	1.00
Donor age at time of transplant	**0.049**	0.004	0.01	0.761	1.00	0.98	1.03

*The univariate analysis defined variables that were significantly associated (*p* < 0.05) or tended to associate (^t^*p* < 0.2) with deterioration of kidney graft function (≥10% eGFR loss 1 year post-enrollment). These variables were used to construct a multivariate Cox regression model to analyze the link between NK cell activation corresponding to the intermediate and high ADCC responses observed in the KTR cohort (CD107a/Lamp1URI ≥ 2.98) and risk of a further 10% decline in allograft function, evaluated in reference to the eGFR values obtained at the time of enrollment and after a median period of 13 months post-enrollment (min–max, 11–41 months post-inclusion). Although found to not be significantly associated with the degradation of kidney graft function using univariate analysis, variables such as the eGFR at the time of NK cell evaluation, the time posttransplant, and the presence of circulating dnDSAs at the time of inclusion were introduced into the multivariate model to control whether the association between NK activation and CKD progression was related to initial graft dysfunction and immunization status*.

The plasma samples obtained during the first round of plasmapheresis treatment from two ABMR patients were used to validate the performance of the NK-CHAT in detecting anti HLA-A2 and HLA-DR4 DSA reactivity. Serum and plasmapheresis samples (500 mL) were collected and aliquoted during the first round of ABMR desensitization therapy from two KTRs completing 10 plasmapheresis sessions, specifically before the initiation of other treatments using pulse methylprednisolone, intravenous immunoglobulin, and rituximab treatment to ensure that rituximab reactivity does not interfere in the experiments conducted with these plasma samples. The first patient (patient 1) was a 59-year-old man with HLA-A2 *dn*DSAs who experienced an acute C4d-positive ABMR rejection 8 years after his first renal transplantation. The second patient (patient 2) was a 29-year-old woman with HLA-DR4 *dn*DSAs who experienced an acute C4d-positive ABMR rejection episode with cellular borderline changes 8 years after her first transplantation. Antibodies from serum or plasma were purified using Protein A columns and Gentle Ag/A binding and elution buffers (Pierce 20356 and 21030). Before introduction into the NK-CHAT assay, the purified antibodies were dialyzed against PBS buffer (2 × 2 h and overnight) using a 10-kDa dialysis cassette.

### Identification of Donor-Specific Anti-HLA Alloantibodies

The detection of HLA-specific antibodies in serum samples was performed using standard techniques. The presence of allograft-specific antibodies was screened through CDC and Luminex screening assays (LAScreen^®^ mixed, One Lambda, Canoga Park, CA, USA) using Luminex flow beads (LAScan™ 100, Luminex, Austin, TX, USA). To determine their antibody specificity, all samples with a positive screening result were further evaluated using “Single-Antigen” Gen-Probe Lifecodes reagents (Lifecodes LSA class I and Lifecodes LSA class II kits, Immucor, Norcross, GA, USA) according to the recommendations of the manufacturer and current guidelines (16, 50). The DSAs present in the samples were analyzed at the time of biopsy and were further characterized using single-antigen flow bead assays according to the manufacturer’s recommended protocol (LAScreen^®^ Single Antigen class I or LAScreen^®^ Single Antigen class II, One Lambda, Canoga Park, CA, USA). Median fluorescence intensity (MFI) values were obtained using the baseline formula proposed by Fusion 3.2 software. The percentage of PRAs for the single-antigen assays were calculated according to the manufacturer’s instructions as the percentage of positive bead reactions among the 99 class I beads and 97 class II beads. Cytotoxic cross-match assays were performed with donor PBMCs or splenic cells according to the protocol recommended by the Eurotransplant Organization using a standard microcytotoxicity assay. The CDC showed positive results when at least 50% of the cells were dead. Autoreaction was detected by incubating the patient’s serum with autologous effector lymphocytes. IgM reactivity was excluded through prior treatment of the tested sera with dithiothreitol (Fluka BioChemika).

### Flow Cytometry Analysis of Antibody Ligation

Antibody ligation to target cells was analyzed using anti-human Fc antibody. The target cells (10^5^) were incubated with 10 μL of the FcR-blocking reagent (Miltenyi 130-059-901) and 90 μL for serum 0 min at 4°C, washed in PBS, incubated with 20 μL of sera or plasma and 80 μL of PBS for 30 min at 4°C, and then washed again in PBS. Binding was evaluated by incubating the serum- or plasma-coated cells with a secondary goat F(ab′)^2^ anti-human-Fc antibody conjugated to PE for 20 min at 4°C (Beckman Coulter IM0550). Acquisition and analysis of the MFI of the gated allogeneic target cells were performed using a Beckman Coulter Navios Cytometer.

### Absorption of HLA Class I Antibodies

Platelets were obtained from the blood bank service as units of platelet concentrate and were processed as follows. First, the platelets were centrifuged at 200 *g* for 40 min in 50-mL centrifuge tubes. The supernatant was removed, and the platelets were centrifuged again at 2,000 *g* for 15 min. After removal of the supernatant, 20 mL of 0.8% ammonium chloride was added to achieve red blood cell lysis, and the mixture was placed on a rotary mixer for 50 min. The platelets were washed twice with 1% Tris-buffered EDTA/saline and stored in a solution containing 0.1% sodium azide until their use for antibody absorption. Prior to absorption, the platelets were centrifuged at 2,000 *g* for 20 min, the supernatant was removed, and the platelets were washed twice with complement fixing buffer (Ovoid). A 50% volume of complement fixing buffer was added to packed platelets. Then, 1 mL of the above-described mixture was placed in a microcentrifuge tube and centrifuged at 10,000 *g* for 5 min, and the supernatant was removed. A volume of 0.25 mL of each sera sample was mixed, incubated at 22°C for 2 h, and centrifuged at 10,000 *g* for 5 min, and the absorption procedure was repeated with an overnight incubation at 22°C. Non-platelet- and platelet-absorbed sera were stored at 4°C until further use.

### Phenotypic Analysis of Antibody-Dependent NK Cell Activation

The NK-CHAT was performed to analyze the antibody-dependent activation potential of NK effector cells resulting from their exposure to rituximab or DSA-coated target cells. Briefly, 500,000 target cells (B-EBV cell lines, NK cell-depleted PBMCs, or spleen cells) were incubated with control (CTL) unsensitized male human AB serum (CTL, Lonza) to block FcRs, rinsed, and incubated for 15 min in the presence of 20% KTR serum or CTL serum either supplemented or not supplemented with 10 μg/mL rituximab or purified IgG. The samples were then rinsed to remove any unbound antibodies. Effector cell PBMCs were incubated with antibody-coated targets for 3 h at 37°C using a 1:1 effector-to-target ratio in the presence of Golgi Stop (Becton Dickinson 554724) and CD107a-PC5 (Becton Dickinson 555802). In several experiments, serum was incubated in the presence of 200 μg/mL of Protein A to block antibody Fc fragment reactivity. The cells were then washed and labeled with CD3-ECD (Beckman Coulter A07748), CD16-PE (Beckman Coulter A07766), and CD56-PC7 (Beckman Coulter A21692) for 15 min at room temperature. Data acquisition and analysis were performed using a Beckman Coulter Navios cytometer. The NK lymphocyte subset within the PBMCs was gated through CD3/CD56-labeling (CD3^−^CD56^+^ population). The CD16 and Lamp1/CD107a expression patterns within the CD3^−^CD56^+^ NK subset were analyzed. ADCC was further analyzed by calculating the rituximab–CD107a/Lamp1 upregulation index (CD107a/Lamp1URI), which is expressed as the ratio between the percentage of CD107a/Lamp1 NK cell activation toward B cells in the presence (ADCC) or absence of rituximab (natural cytotoxicity). The level of CD16 engagement was quantified as the ratio between the MFIs of NK CD16 expression observed after effector PBMCs were incubated with B cell targets exposed to 20% human CTL DSA^−^ serum in the presence or absence of rituximab and was further defined as the CD16 downregulation index (CD16DRI).

When PBMCs or splenic cells were used as the target cells, the cell preparations were depleted of NK cells prior to being used as targets in the NK-CHAT. NK cell depletion was achieved through CD16 and CD56 magnetic bead selection (Miltenyi 130-045-701 and 130-050-401). Briefly, pelleted cells were resuspended in 60 μL of PBS containing 2 mM EDTA, 0.5% human AB serum (SAB), and 20 μL of each set of beads and then incubated on ice for 30 min. After a wash step, the fixed NK cells were depleted using adapted columns and magnets as recommended by the manufacturer (Miltenyi 130-042-201).

### Transplant Histological Assessment

Forty transplant biopsies were retrospectively reassessed and scored using the updated conventional Banff diagnosis criteria (51) by one experimental renal pathologist (LD) blinded to the clinical information. C4d staining of paraffin section was performed through immunochemistry (51, 52). The biopsies were graded (from 0 to 3) according to the following histological parameters: glomerulitis (g), tubulitis (t), interstitial inflammation (i), intimal arteritis (v), peritubular capillaritis (ptc), chronic glomerulopathy (cg), interstitial fibrosis and tubular atrophy (ci and ct), and arterial fibrous intimal thickening (cv). The humoral parameters were integrated into a humoral histological score by adding the following variables: (g + ptc + v + cg + C4d) (36).

### Statistical Analysis

The statistical analyses were performed using Graph Pad Prism 5 software (GraphPad Software, La Jolla, CA, USA) and IBM SPSS Statistics for Windows, Version 20.0 (IBM Corp., Armonk, NY, USA). Categorical variables are reported as counts or percentages. The associations between continuous variables were analyzed using Spearman’s rank correlation analysis. The Chi-square test (or Fisher’s exact test when appropriate) was used for the comparisons of categorical variables. Group comparisons were performed through one-way analysis of variance (ANOVA). A Mann–Whitney *t* test was performed for the comparison of non-parametric data from two groups. A *p* value <0.05 was considered to represent a statistically significant difference. In all figures, one asterisk (*) denotes *p* < 0.05, two asterisks (**) denote *p* < 0.01, and three asterisks denote *p* < 0.001. Clinical, histological, functional, and immunological factors associated with the degradation of kidney graft function were assessed in a separate univariate analysis. The variables identified as significantly associated with outcome variables (*p* < 0.05), that were marginally significant (*p* < 0.20, *t*) in the univariate analysis, or that are considered clinically relevant were selected for inclusion in the Cox regression model, which analyzed parameters associated with a loss in kidney graft function equal to at least 10% of the eGFR over the follow-up period of the 148 patients (median duration of follow-up: 13.3 months, 25–75th percentile: 12.1–15.1 months post-evaluation).

## Results

### Analysis of Antibody-Dependent NK Cell Responses in Kidney Transplant Recipients

**Figure 1:**
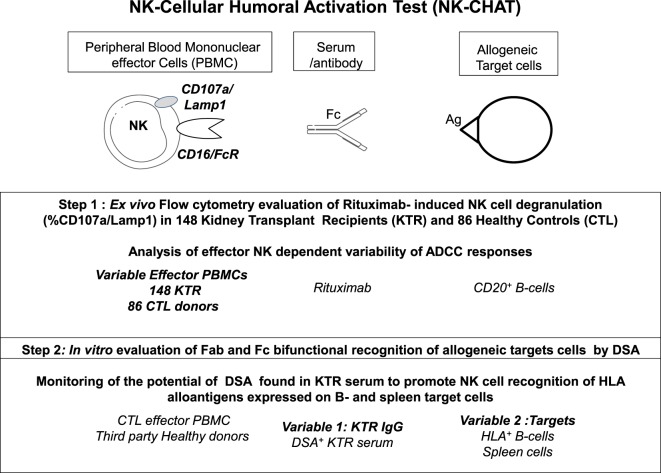
**Description of the observational and *in vitro* steps of the study of antibody-dependent NK cell activation using a standardized NK-Cellular Humoral Activation Test (NK-CHAT)**. Step 1. The standardized *ex vivo* NK-CHAT evaluation of the variability of KTR NK cell activation toward B cell targets coated with anti-CD20 monoclonal antibodies (rituximab). Step 2. *In vitro* scoring of serum DSA activity toward allogeneic target cells using third-party PBMCs isolated from healthy blood donors as immune effector cells.

**Figure 2:**
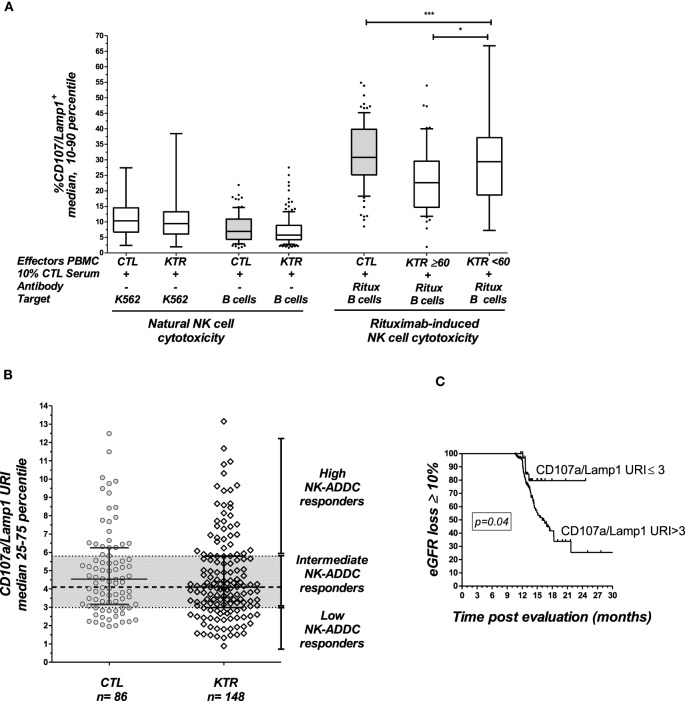
**Analysis of NK cell activation in late kidney transplant recipients**. **(A)** The natural cytotoxicity of NK cells toward HLA-negative K562 cells or CD20^+^ B-lymphocyte target cells was analyzed by multicolor flow cytometry. The expression of CD107a/Lamp1 on the surface of CD3^−^CD56^+^ NK cells gated within the PBMCs was analyzed. NK cell ADCC toward the same B cell targets was evaluated in the presence of rituximab (Ritux). NK cell activation was evaluated in 148 KTRs and 86 healthy controls (CTL). The KTR patients were grouped according to graft function as follows: normal graft function (eGFR ≥ 60 mL/min/1.73 m^2^, *n* = 56) and moderate-to-severe graft dysfunction (eGFR < 60 mL/min/1.73 m^2^, *n* = 92). One-way ANOVA was performed to test the significance of NK-ADCC using PBMC effector cells obtained from CTLs and the KTR subgroups (Kruskal–Wallis, *p* = 0.0002). Asterisks indicate the *p* values as follows: *p ≤ 0.05, **p ≤ 0.01, and ***p ≤ 0.001. The box plots show the median values, and the whiskers represent the 5–95 percentiles. **(B)** Specific analysis of rituximab-induced NK cell activation. To analyze the specific contribution of antibodies, NK cell activation toward rituximab-coated B cells was normalized in reference to the baseline cytotoxicity of the NK cells toward B cells evaluated in the absence of rituximab by calculating the CD107a/Lamp1 upregulation index (CD107a/Lamp1URI). The CD107a/Lamp1URI values observed in the KTR cohort allowed classification of the KTRs as low ADCC responders if their CD107a/Lamp1URI was below 3 (25th percentile) or as intermediate or high responders if their CD107a/Lamp1URI value was greater than 3. **(C)** Kaplan–Meir curves for an eGFR loss of at least 10% or graft loss (*n* = 51) observed during the follow-up period (months post-inclusion in the study) in KTRs with low NK cell activation (CD107a/Lamp1URI ≤ 3) and intermediate or high NK cell activation (CD107a/Lamp1URI > 3).

The NK-CHAT assay was designed to evaluate the contribution of the three components of ADCC responses, i.e., NK effector cells, antibodies, and target cells (Figure 1). In the first approach, peripheral NK cell cytotoxic activity was evaluated using a standardized combination of targets and antibodies, and variable sources of circulating effector cells isolated from 148 late KTRs were compared to those from 86 healthy controls (Table 1). Flow cytometry analysis of CD107a/Lamp1^+^ NK cell granule exocytosis allowed quantification of natural and antibody-dependent cytotoxic activation against a standardized combination of HLA-negative (K562) target cells or HLA- and CD20-positive B cell target cells evaluated in the presence or absence of rituximab. As observed for the control cells, the NK cell cytotoxic function was highly variable in KTRs (Figure 2A). The variations in the levels of NK cell cytotoxic activation toward K562 (*p* = 0.206) or B cell target cells (*p* = 0.141) were not significantly different between the KTR and control groups. Interestingly, although the ADCC levels of KTRs with an eGFR < 60 mL/min/1.73 m^2^ were comparable to those observed in the control group, the NK cell ADCC responses were significantly decreased in transplant recipients with preserved graft function (eGFR > 60 mL/min/1.73 m^2^). To normalize the interindividual variability of natural NK cell cytotoxicity toward B cells, NK-ADCC was further indexed by calculating the rituximab–CD107a/Lamp1 upregulation index (CD107a/Lamp1URI), a ratio of the percentage of CD107a/Lamp1^+^ NK cell activation toward B cells in the presence to that observed in the absence of rituximab (Figure 2B). Although highly variable, the level of rituximab-induced CD107a/Lamp1 upregulation was maintained across KTRs (median CD107a/Lamp1URI: 4.1, 25–75th percentile: 2.98–5.79) and was not significantly different from that observed in a cohort of healthy control individuals (median CD107a/Lamp1URI: 4.5, 25–75th percentile: 3.16–6.25, Figure 2B). A multivariate Cox regression analysis of the factors associated with CKD progression further indicated that this intermediate level of the ADCC responsiveness observed in KTRs (CD107a/Lamp1URI > 3) was associated with the occurrence of graft function decline (≥10% eGFR loss) or graft failure in 34% of the transplanted recipients during the mean follow-up period of 13.3 months (Figure 2C and Table 2).

### Quantification of DSA-Dependent CD16 Engagement and NK Cell Activation toward Allogeneic Cell Targets

**Figure 3:**
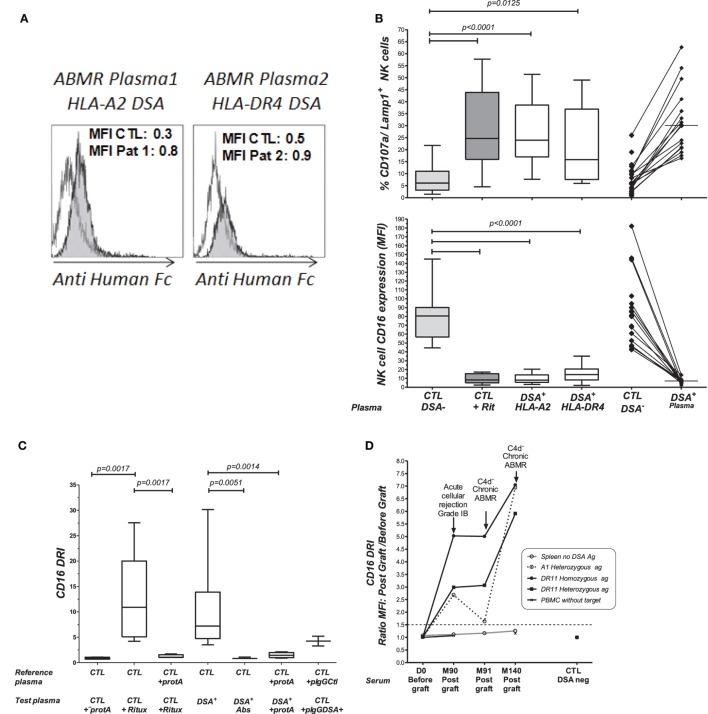
**Comparative analysis of rituximab and DSA-specific NK cell activation**. **(A)** Plasmapheresis samples were obtained during plasma exchange therapy from two patients with circulating DSAs and a biopsy-proven ABMR. The binding of anti HLA-A2 (patient 1, Pat1) or anti HLA-DR4 DSA (patient 2, Pat2) to B cell targets was revealed by flow cytometry cross-match (FCMX) using a secondary antibody (gray shaded area). FCXM was analyzed in reference to binding and was observed when B cells were coated with the control plasma, showing no humoral reactivity (white area). MFI, median fluorescence intensity. **(B)** Comparative analysis of rituximab and DSA-induced NK cell activation toward target cells expressing HLA alloantigens. Flow cytometric analysis of antibody-mediated CD107a/Lamp1 (upper panel) and CD16 (MFI) (lower panel) expression was performed using NK cells prepared from distinct third-party healthy donors after incubation with HLA-A2 homozygous and DR4 heterozygous B cell targets and HLA-A2 DSA^+^ plasma (*n* = 17) or HLA-DR4 DSA^+^ plasma (*n* = 11). CTL, control plasma with no detectable anti HLA antibodies. **(C)** NK-CHAT analysis of DSA^+^ plasma. CD16DRI was evaluated using effector cells exposed to B cell targets in the presence of rituximab, DSA^+^ plasma, or DSA^−^ CTL plasma. The specificity of antibody-driven CD16 engagement was analyzed by the addition of Protein A (53), by prior platelet absorption (Abs) of the anti-HLA antibodies in plasma, or through the use of IgG purified from ABMR plasma (pIgG DSA^+^) or control plasmas with no detectable HLA antibodies (pIgG CTL). **(D)** CD16DRI was used to evaluate the reactivity of serum samples obtained during the follow-up period of KTRs toward four different allogeneic splenic cells from the day of transplant (D0) to months (M) 90, 91, and 140 after transplant. The target splenic cells were selected according to the presence or absence of HLA antigens recognized by anti HLA-A1 or HLA-DR11 DSAs: (o) no relevant HLA antigen, (---) HLA-A13 heterozygote, and (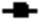) HLA-DR11 homozygous (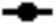) or HLA-DR11 heterozygous antigens. An enhanced CD16DRI of NK cells that were exposed to splenic cells expressing DSA cognate HLA alloantigen was obtained using serum obtained at M90 from this KTR patient at the time of acute cellular rejection diagnosis. Persistent elevated CD16DRI levels were obtained using serum obtained at the times of two consecutive episodes of C4d^−^ diagnosis of chronic ABMR, which occurred at M91 and M140 after transplant. The values of CD16 MFI obtained when effector PBMCs exposed to serum in the absence of splenic cells served as controls for baseline activity (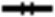).

Through the introduction of KTR serum as a variable in the NK-CHAT assay, we further investigated whether this test could assess the variability in DSA-mediated NK cell cytotoxic activity, which relies on the structural features of IgGs found in complexes in the KTR serum (Figure 1, step 2). Consistent with studies that utilized C1q binding as a tool to characterize the complement-dependent pathogenicity of DSA, we explored whether indexing the Fc–FcR interactions can reflect the cytotoxic potential of DSAs found in plasmapheresis samples collected during ABMR therapy. Specific binding of the DSAs found in plasma to B cells expressing either HLA-A2 or DR4 antigen was confirmed by flow cytometry cross-match (FCXM, Figure 3A). Plasma containing anti-HLA-A2 DSAs induced a significant and reproducible modulation of both CD107a/Lamp1 and CD16 expression on NK cells incubated with distinct HLA-A2^+^ B cell targets (Figure S1A in Supplementary Material). A phenotypic analysis of NK cells identified CD16 expression as a surrogate marker of NK cell cytotoxic granule exocytosis and shows that it has the capacity to reflect the specific humoral component of NK cell activation (Figure 3B). A comparative NK-CHAT evaluation of DSA^+^ plasma samples and rituximab reactivity was performed using different batches of effector PBMCs from distinct healthy donors (*n* = 25). The NK-CHAT scores resulting from the specific recognition of allogeneic B cells by DSAs were not significantly different from those observed with rituximab (Figure 3B). As observed with rituximab, the plasma-driven modulation of CD107a/Lamp1 and CD16 expression was specific, exhibiting HLA alloantigen recognition and effector-dependent variability (Figure 3B). The same results were observed using freshly isolated NK cell-depleted human PBMCs as the allogeneic target cells expressing HLA alloantigens (Figure S1B in Supplementary Material). To normalize the variability in the baseline expression of CD16 in KTRs, a CD16 downregulation index (CD16DRI) was calculated as the ratio of the CD16 MFI measured in response to B cells coated with DSA-negative control serum (baseline) to the CD16 MFI results from B cells coated with KTR sera. In most cases in which the same sources of effector and target B cells were used in the NK-CHAT assay, the CD16DRI values measured in response to DSA-coated cells were correlated with the CD16DRI values measured in response to rituximab-coated cells (Spearman *r* = 0.7, *p* < 0.0001).

Antibody-mediated CD16 engagement was partially inhibited by Fc blockage using Protein A and by prior removal of DSAs from the serum. Specific CD16 engagement *via* the Fc fragment of DSAs was also observed after the exposure of target B cells to IgG purified from DSA^+^ plasma samples (Figure 3C).

The analysis of the use of NK cell-depleted splenic cells, a common source of donor cells used in cross-match assays, as cell targets in the NK-CHAT showed that CD16 engagement also reflected the anti-HLA-A2 DSA-specific recognition of donor cells (Figure S2 in Supplementary Material). We used different batches of splenic cells to monitor the NK-CHAT reactivity of the pre- and posttransplant serum samples obtained from a patient who developed HLA-A1 and DR11 *dn*DSAs associated with graft dysfunction and consecutive ABMR episodes (Figure 3D). The CD16DRI values evaluated against HLA-DR11 homozygous spleen cells were higher than those against DR11 heterozygous spleen target cells. Interestingly, in this patient, the increased CD16DRI value was associated with the progression of histological lesions consistent with C4d–negative ABMR.

### NK-CHAT Evaluation of Serum Sampled at Time of Biopsy-Proven ABMR Diagnosis

**Figure 4:**
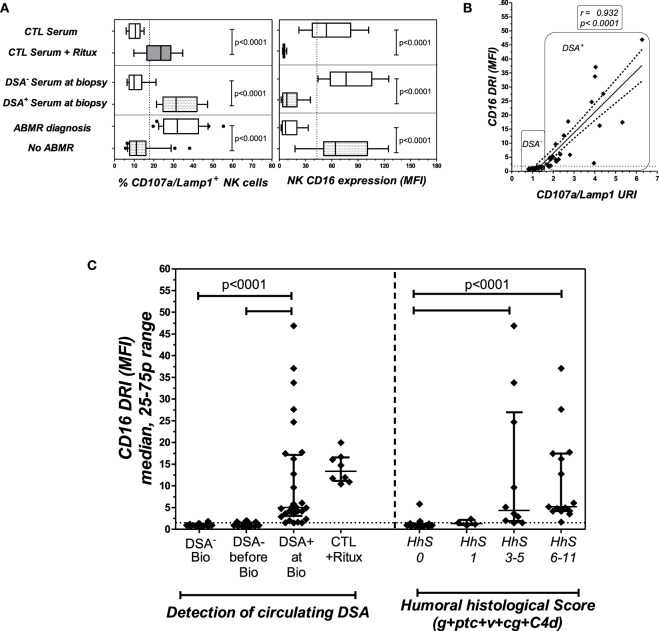
**The NK-CHAT evaluation of DSA reactivity toward B cell targets in sera collected at the time of transplant biopsy**. **(A)** The modulation of CD16 (MFI) and CD107a/Lamp1 on NK effector cells exposed to serum-coated B cell targets was analyzed. Forty-three sera samples were collected from 40 KTRs at the time of biopsy and grouped according to the detection (DSA^+^, *n* = 25 sera obtained in 22 patients) or absence (DSA^−^, *n* = 18 patients) of B cell-specific circulating DSAs (the DSA specificities and MFI are detailed in Table 4) or to ABMR diagnosis. Rituximab was added to DSA^−^ serum and used as a positive control for the test. **(B)** Correlation of CD16DRI and CD107a/Lamp1URI scores for sera samples collected at the time of biopsy. **(C)** At the time of transplant biopsy (Bio), higher CD16DRI scores were associated with the presence of circulating DSAs (left panel) and humoral histological scores greater than 3 (g + ptc + v + cg + C4d of Banff’s classification, right panel). The CD16RI scores of DSA^−^ serum from the same sensitized KTR, obtained prior to the detection of *dn*DSAs (before Bio), were also evaluated in reference to DSA^+^ serum sampled at the time of biopsy.

To investigate the potential link between CD16DRI and DSA-mediated allograft injury, the NK-CHAT was performed using 46 sera samples obtained from 40 KTRs who had undergone concomitant transplant biopsies for clinical evaluation (Table 3). Among these KTRs, 18 patients were not sensitized, whereas the sera collected from 22 patients showed detectable levels of *dn*DSAs (HLA specificities listed in Table 4). The serum-driven modulation of the CD16 and CD107a/Lamp1 levels was associated with the detection of circulating DSAs and the histological diagnosis of ABMR (Figure 4A). The CD16DRI values were positively correlated with CD107/Lamp1URI (Figure 4B) and varied among DSA^+^ sera (Figure 4C). Although anti-DQ7 DSAs with similar MFI values sometimes exhibited variable levels of CD16DRI (Table 4, illustration in Figure S3 in Supplementary Material), the median CD16DRI values were found to correlate with the MFI intensity of DSAs detected by Luminex assays (Spearman *r* = 0.46, *p* = 0.016). For patients with DSAs and ABMR, histological scores (g + ptc + v + cg + C4d) above 3 were associated with a higher CD16DRI value (Figure 4C). In particular, the NK-CHAT was sufficiently sensitive to index the reactivity of an anti-Cw04 DSA in a patient with an ABMR-related microangiopathy (Table 4, patient 19). Monitoring of the pre- and posttransplant sera from 18 of these *de novo* sensitized patients revealed that NK activation was only detected posttransplant, specifically at the time of *dn*DSA detection (Figure 4C), thus allowing evaluation of the CD16DRI values in reference to the pretransplant sera.

**Table 3:** **Characteristics and histological Banff scores of the 40 patients subjected to evaluation of the serum CD16DRI obtained at the time of histological diagnosis**.

Parameters	Patients with DSA directed against B cell targets (*n* = 22)	Patients without DSA (*n* = 18)	*p* value
Recipient age at biopsy (years)	52.1 (15)	51.4 (15.4)	ns
Male	13 (59%)	13 (72%)	ns
Preemptive graft	1 (4%)	1 (6%)	ns
**Risk factors for HLA sensitization**			
Second transplantation	2 (9%)	0	ns
Blood transfusion before graft	9 (41%)	7 (39%)	ns
HLA A+ mismatch	2.3 (1)	2.5 (0.9)	ns
HLA DR mismatch	1.0 (0.6)	1.0 (0.7)	ns
Deceased donors	19 (86%)	18 (100%)	ns
Donor age (years)	37.2 (17)	46.1 (17.1)	0.11
Expanded criteria donors	4 (18%)	5 (28%)	ns
Delayed graft function	2 (9%)	3 (17%)	ns
Time since transplantation (Mo)	124 (72)	62 (69)	0.008
**Indication for biopsy**			
Deterioration of graft function	20 (91%)	14 (78%)	ns
Investigate proteinuria	2 (9%)	1 (5%)	ns
BK virus viremia	0	3 (17%)	0.08
**Maintenance immunosuppressive regimen at biopsy**			
Tacrolimus	10 (45%)	9 (50%)	ns
Cyclosporine	12 (54%)	9 (50%)	ns
MMF	10 (45%)	8 (44%)	ns
Azathioprine	3 (14%)	7 (39%)	0.06
Steroid	17 (77%)	18 (100%)	0.03
**Graft function at biopsy**			
Serum creatinine (μmol/L)	230 (80)	200 (66)	0.13
eGFR (mL/min/1.73 m^2^)	28 (10)	35 (15)	0.15
Proteinuria (g/L)	1.0 (0.20–2)	0.45 (0.10–0.80)	0.04
**Panel-reactive antibody at biopsy**			
Class I	9% (19)	0%	0.004
Class II	16% (14)	0%	<0.0001
**Histological findings**			
Sclerotic glomeruli (%)	33%(23)	26%(24)	ns
g score (0–3)	0.9	0	0.0004
ptc score (0–3)	1.8	0.1	<0.0001
Microcirculation inflammation (g + ptc)	2.7	0.1	<0.0001
g + ptc > 0	19	2	<0.0001
*V* score (0–3)	0.2	0	ns
cg score (0–3)	1.1	0	0.0004
IF/TA (0–3)	1.7	1.5	ns
cv score (0–3)	1.6	1.1	0.01
Humoral histologic score (g + ptc + v + cg + C4d)	6 (4–8)	0	<0.0001
T-cell-mediated rejection	7 (32%)	3 (17%)	ns
Antibody-mediated rejection	20 (91%)	0	<0.0001
**NK cell activation**			
CD107a/Lamp1 URI median (25–75p)	2.1 (1.9–3.9)	1.1 (0.99–1.3)	<0.0001
CD16 DRI median (25–75p)	5 (3–17)	0.96 (0.8–1.16)	<0.0001

*The sera samples were classified into two groups according to the presence or absence of DSAs directed against B cell targets. g, glomerulitis; ptc, peritubular capillaritis, IF/TA, interstitial fibrosis/tubular atrophy. The variables are shown as the means (SD), medians (25–75th percentile), or *n* (%). χ^2^ tests were used for comparisons of the proportions, and unpaired *t* tests or Mann–Whitney tests were used for the comparisons of continuous variables*.

**Table 4:** **PRA and MFI of 25 DSA^+^ serum samples subjected to evaluation of the CD16DRI score at the time of biopsy**.

KTR serum	PRA CLI	PRA CLII	Circulating DSA	Specific MFI toward HLA alloantigens target B-cell 2	A2	A2	B44	B56	Cw1	DR1	DR4	DR53	DQ5	DQ7	Median CD16DRI (*n* = 2–6)
*Pat. 01 – S01*	14	33	A3 A29 B56 DR11 **DQ7**	*23,000*	0	0	0	0	0	0	0	0	0	**23,000**	17.7
*Pat. 02 – S02*	15	20	B44 **DQ5**	*9,500*	0	0	0	0	0	0	0	**3,500**	**6,000**	0	2.0
*Pat. 03 – S03*		13	**DQ5**	*15,000*	0	0	0	0	0	0	0	0	**15,000**	0	3.5
*Pat. 04 – S04*		6	DR12 **DQ7**	*12,000*	0	0	0	0	0	0	0	0	0	**12,000**	4.2
*Pat. 05 – S05*	35		**DR53**	*12,000*	0	0	0	0	0	0	0	12,000	0	0	5.8
*Pat. 06 – S06*		21	**DQ7**	*13,000*	0	0	0	0	0	0	0	0	0	**13,000**	33.8
*Pat. 07 – S07*		9	**DQ7**	*2,000*	0	0	0	0	0	0	0	0	0	**2,000**	1.7
*Pat. 08 – S08*		21	**DQ7**	*15,000*	0	0	0	0	0	0	0	0	0	**15,000**	6.0
*Pat. 09 – S09 Bio 1*		18	**DQ7**	*9,000*	0	0	0	0	0	0	0	0	0	**9,000**	24.7
*Pat. 09 – S09 Bio 2*		22	**DQ7**	*15,000*	0	0	0	0	0	0	0	0	0	**15,000**	37.1
*Pat. 10 – S10 Bio 1*	37		**A2, B44**	*20,000*	8,000	8,000	4,000	0	0	0	0	0	0	0	3.6
*Pat. 10 – S10 Bio 2*	34		**A2, B44**	*5,000*	1,500	1,500	2,000	0	0	0	0	0	0	0	1.7
*Pat. 10 – S10 Bio 3*	90		**A2, B44**	^a^	^a^	^a^	^a^	0	0	0	0	0	0	0	5.2
*Pat. 11 – S11*	5	5	A1 **DR53**	*10,000*	0	0	0	0	0	0	0	**10,000**	0	0	12.7
*Pat. 12 – S12*		20	**DQ5**	*10,000*	0	0	0	0	0	0	0	0	**10,000**	0	4.2
*Pat. 13 – S13*	20	20	**A2 B44 DQ5**	*24,000*	**2,800**	**2,800**	**7,500**	0	0	0	0	0	**14,000**	0	5.1
*Pat. 14 – S14*		20	**DR53 DQ7**	*19,000*	0	0	0	0	0	0	0	**9,000**	0	**10,000**	27.6
*Pat. 15 – S15*	15	10	A2 DQ5	*32,000*	14,000	14,000	0	0	0	0	0	0	4,000	0	17.4
*Pat. 16 – S16*		23	DQ7	*14,000*	0	0	0	0	0	0	0	0	0	14,000	46.9
*Pat. 17 – S17*	33	5	B56 DR4	*12,500*	0	0	0	10,000	0	0	2,500	0	0	0	16.2
*Pat. 18 – S18*		1	**DR1**	*500*	0	0	0	0	0	**500**	0	0	0	0	2.9

				**Specific MFI toward HLA alloantigens Target B-cell 2**	**A3**	**A3**	**B7**	**B35**	**Cw4**	**DR10**	**DR15**	**DR51**	**DQ5**	**DQ6**	

*Pat. 19 – S19*	15		**Cw4**	9,500	0	0	0	0	**9,500**	0	0	0	0	0	4.8
*Pat. 20 – S20*	15	3	**A3** A26 C7 **DQ6**	16,000	**5,000**	**5,000**	0	0	0	0	0	0	0	**6,000**	9.6
*Pat. 21 – S21*		60	**DQ6**	15,000	0	0	0	0	0	0	0	0	0	**15,000**	1.5
*Pat. 22 – S22*		19	DR17 **DQ6**	3,000	0	0	0	0	0	0	0	0	0	**3,000**	2.3
*Pat. 01 – S01*	14	33	A3 A29 B56 DR11 **DQ7**	10,000	**5,000**	**5,000**	0	0	0	0	0	0	0	0	4.2
*Pat. 02 – S02*	15	20	B44 **DQ5**	10,000	0	0	4,000	0	0	0	0	0	**6,000**	0	1.5
*Pat. 03 – S03*		13	**DQ5**	15,000	0	0	0	0	0	0	0	0	**15,000**	0	4.9

*A total of 25 DSA^+^ sera samples obtained from 22 KTR patients (Pat.) at the time of histological diagnosis were evaluated by CD16DRI scoring. Two distinct B cell targets (HLA genotype-indicated) were used to detect HLA alloantigen recognition. Sera obtained from patients 1–3 exhibited DSA^+^ reactivity toward both target B cells, and tests were evaluated on the two cell targets. The CD16DRI scores were evaluated through at least two independent experiments (*n* = 2–6), and the presented CD16DRI values are the medians of these tests*.

*HLA specificities and specific MFI of circulating donor specific antibodies (DSA) towards target alloantigens expressed on B cell lines 1 and 2 are indicated in bold characters*.

*^a^The circulating DSAs evaluated in Pat. 10 at the time of graft nephrectomy showed very high titers, which did not enable accurate MFI quantification. HLA class I (CLI) or class II (CLII) panel-reactive antigens (PRA) are indicated as percentages*.

**Figure 5:**
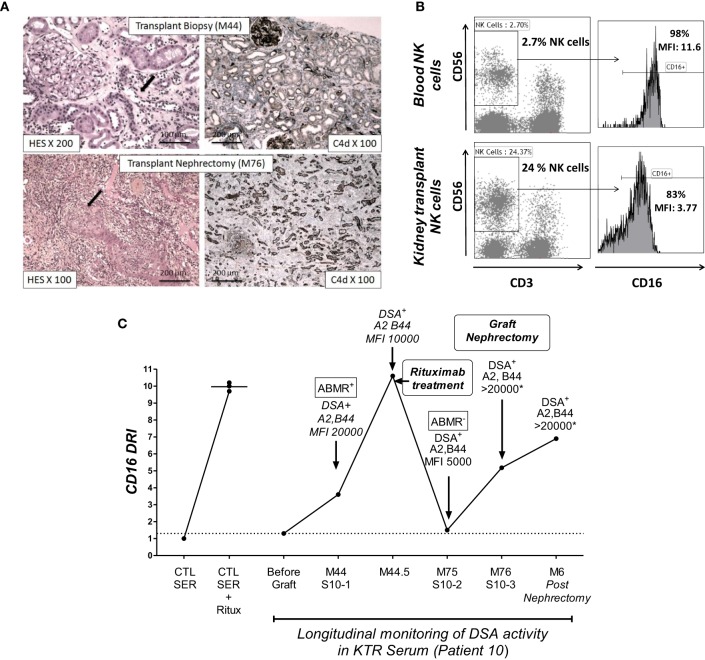
**The NK-CHAT monitoring of DSA reactivity and characterization of CD16 engagement of NK cells infiltrating a kidney transplant site**. **(A)** Histological assessment of lesions from biopsies of patient 10 at M44 and M76 (transplant nephrectomy). The M44 histological analysis showed peritubular capillaritis (cpt = 2, black arrow). At M76, histology revealed arteritis (v = 2, black arrow) and confirmed severe acute inflammatory lesions (t = 3, i = 3, g = 3, ptc = 3, v = 2, C4d = 3) with diffuse interstitial edema and chronic vascular lesions (cv = 2) as well as tubular atrophy (ct = 2). The left and right panels show the hematoxylin–eosin–saffron (HES) and C4d immunochemical staining of paraffin sections, respectively. **(B)** The NK-CHAT activity of five sera samples obtained during the longitudinal monitoring of patient 10 was analyzed in reference to serum obtained before transplantation (no sensitization). A significant increase in CD16DRI was concomitant with the detection of HLA-A2 and anti-B44 *dn*DSA at M44 as well as histological lesions of ABMR. The CD16DRI level decreased after rituximab treatment. The detection of low levels of circulating DSAs at M75 (before graft nephrectomy) was associated with a lowered CD16DRI and no evidence of humoral activity within the biopsy. An increased CD16DRI was observed at the time of graft nephrectomy and persisted for 6 months. At these two time points (M76 and 6 months after graft nephrectomy), the circulating DSA were at saturating titers, which did not allow accurate MFI quantification. **(C)** Comparative flow cytometric analysis of CD16 expression in blood CD3^−^CD56^+^ NK cells and infiltrating NK cells isolated from the transplant after nephrectomy.

To illustrate the ability of the NK-CHAT to monitor the evolution of DSA reactivity and the potential relevance of NK cell activation during humoral rejection, we evaluated a patient experiencing her first acute ABMR attack at month 44 with subsequent graft loss. The NK-CHAT analysis revealed a significant increase in the CD16DRI value associated with biopsy-proven ABMR (Figure 5A). Moreover, the CD16DRI value decreased after ABMR treatment (plasmapheresis and rituximab) but was markedly increased at the time of graft failure. A transplant nephrectomy was performed at month 76 due to graft intolerance syndrome (Figure 5B). A comparative phenotypic analysis of circulating and kidney-infiltrating NK cells showed that the NK cells constituted 24% of the intra-graft lymphocytes, whereas only 2.7% of the peripheral blood lymphocytes at the time of transplant nephrectomy were NK cells. When analyzed in reference to circulating NK cells, a sixfold decrease in the CD16 MFI was also observed in the intra-graft NK cells, suggesting that the *dn*DSA-mediated engagement of the CD16 receptor detected by the NK-CHAT may reflect the level of humoral *in situ* NK cell activation within the transplant site (Figure 5C).

## Discussion

Our study highlights the potential value of NK cell evaluation in monitoring the deleterious effects of alloantibodies in solid organ transplantation. Our observational study shows that the intensity of NK cell activation, evaluated through a standardized assay in response to a monoclonal therapeutic IgG, such as rituximab, may be associated with the progression of late graft dysfunction in KTRs. In addition, our results demonstrate the potential of the NK-CHAT to individualize the intrinsic capacity of an individual KTR to mount deleterious humoral immune cytotoxic responses against the graft, independently of other factors that are expected to impact NK cell and transplant function (step 1, Figure 1). The patients with the poorest transplant prognoses 1 year after analysis were predominantly those that exhibit at least a threefold increase in ADCC reactivity in response to rituximab. Our observations indicate that, independently of the detection of circulating DSAs, the individualized NK-CHAT scoring of peripheral NK cell activity may provide relevant information concerning complement-independent mechanisms that favor the development of chronic allograft injury. Because the evaluation of KTRs was only previously performed at one time point years after transplant, we cannot exclude the possibility that subclinical levels of DSAs, which may have developed prior to evaluation, had already promoted chronic NK cell activation *in vivo*, thus restraining the capacity of NK cells from sensitized patients to further respond to rituximab. A recent study demonstrated that pre-ligation of CD16 by anti-CD20 antibodies could impair antibody-mediated NK cell cytotoxicity against tumors (54), thus highlighting the exquisite plasticity of CD16^_^mediated NK cell responses. If translated into a simplified test, we expect that such individualized appraisal of the NK-ADCC activation potential may warrant closer humoral monitoring and recommend biopsy for a subset of immunized patients with high ADCC responsiveness, whereas less stringent monitoring of DSAs may be considered in patients with lower ADCC reactivity. This variability in KTRs regarding NK cell responses also suggests that the NK-CHAT may also be of value for anticipating or monitoring the efficiency of rituximab treatment in sensitized patients (55, 56).

Consistent with mechanistic evidence showing that NK cells play a critical role in mediating long-term transplant kidney injury (29), such intrinsic variability in the NK-ADCC responsiveness is also expected to condition the level of DSA toxicity *in vivo* (24–30). Consistently, we observed that NK cells infiltrating the graft of a KTR with ABMR exhibited enhanced *in situ* CD16 engagement compared with peripheral NK cells.

In the second part of our study, we obtained *in vitro* evidence that the NK-CHAT may also act as a powerful tool to discriminate the differential ability of DSAs to stimulate CD16-dependent NK cell activation (step 2, Figure 1). One unique feature of the NK-CHAT is its potential to combine the analysis of two parameters that control the strength of the target/effector cell interactions *via* DSAs through the following mechanisms: (i) Fab recognition of *ex vivo* conformational antigens and (ii) Fc structural changes. Because a strong CD16DRI was shown to reflect the humoral component of NK cell cytotoxic activation in response to DSA, we expect that the NK-CHAT evaluation could be limited to the measurement of serum-induced CD16 expression.

The scoring of DSA-mediated NK cell activation was not restricted to B cell targets expressing high levels of HLA antigens but was also performed toward peripheral PBMCs and spleen cell targets that express lower physiological levels of HLA antigens. The NK-CHAT also detected functional immune activation resulting from less commonly evaluated alloantibodies, such as the anti-HLA-Cw and DQ7 antibodies. Our observations suggest that the sensitivity of the NK-CHAT to evaluate the ability of DSAs to bind to alloantigens could be comparable or even superior to that of current methods used to characterize DSAs.

A major feature of the NK-CHAT is its potential to detect Fc-dependent variations in alloantibodies and index their potential to trigger CD16-mediated NK cell activation in an individual KTR. Interestingly, DSAs with a comparable MFI against DQ7 alloantigens were, in some cases, associated with different ranges of CD16DRI values. Thus, our test may also reflect structural features of DSAs, which cannot be fully revealed by a SABA. The efficiency of FcR-mediated NK cell activation has been reported to be dependent on the IgG1/IgG3 subclasses (57) and on the glycosylation/sialylation status of the Fc fragments within these antibodies (58). Altered fucosylation patterns in the Fc tails of DSAs have been shown to alter Fc binding to FcγRIIIA and C1q (59) and may represent a critical regulatory determinant of both ADCC- and complement-dependent DSA toxicity (60). The CD16 expression levels and polymorphisms have also been shown to influence NK cell activation (40, 57). Although the KTR CD16 genotype was not included in the initial study design, we expect that depending on the FcγRIIIA genetic background of the recipient, CD16-mediated NK-CHAT responses may be differentially triggered by IgG based on the presence of a valine or phenylalanine residue at position 158 of the FcγRIIIA receptor.

Although the NK-CHAT was mostly associated with the results of the C4d histological staining of the biopsy, we demonstrated that the NK-CD16DRI-based monitoring of DSA activity toward splenic cells was associated with the progression of ABMR lesions that occurred in the absence or prior to the detection of C4d histological staining. A longitudinal follow-up of serum reactivity against allogeneic targets revealed that the NK-CHAT scores were lowered after ABMR desensitization therapy and were associated with the progression of biopsy-proven graft lesions.

Altogether, the results of our study provide evidence that independently of the DSA MFI intensity, the NK-CHAT reactivity may serve as a hallmark of clinically relevant features that discriminate the ability of DSAs to target allograft injury.

Our work has several limitations. First, we only analyzed the degradation of eGFR in 148 KTRs, and the ability of the NK-CHAT to predict kidney dysfunction needs to be demonstrated through CD16 monitoring in a larger cohort of KTRs and over a longer follow-up period. We also need to challenge our test with more stringent end-points, such as graft failure and patient mortality. Considering the major role of complement in the pathogenesis of ABMR, the absence of DSA C1q and C3d-binding tests and the use of complement-depleted serum constitute additional limitations. Moreover, the NK-CHAT scores were obtained using sera that were collected at the time of the clinically indicated transplant biopsy, and the value of the test requires control testing of sera that develop *dn*DSAs in the absence of allograft rejection or exhibit signs of vascular lesions in the absence of detectable levels of DSAs. Due to a low number of cases, the establishment of a firm link between the intensity of NK cell activation and the severity of ABMR lesions or clinical outcome was not possible at this stage. Therefore, it may be relevant to investigate whether the NK-CHAT only serves as a powerful detector of DSAs or if it could predict subclinical ABMR in a cohort of KTRs developing *dn*DSAs as well as anticipate the evolution and severity of ABMR histological lesions.

Despite these limitations, we provide novel evidence of the role of NK cells in renal allograft dysfunction and tools to monitor the interindividual variability of humoral alloimmune responses in immunized patients. A full use of the NK-CHAT using a combination of recipient NK cells and serum combined with donor target cells should improve the ability to identify immunized patients who are at higher risk of subsequent graft dysfunction. Our study highlights the importance of designing and translating novel assays that combine the simultaneous evaluation of the recipient IgG and FcR to individualize the evaluation of the humoral risk of a given recipient. In particular, we developed a simple phenotypic assay that integrates both environmental and genetic parameters that condition antibody–antigen interactions combined with FcR engagement (43, 45). Because this non-invasive assay of CD16 engagement was shown to be reproducible, inexpensive, and easy to implement for routine monitoring, prospective studies are warranted to assess the clinical relevance of the NK-CHAT using recipient NK cells and serum reactivity against target cells of donor origin.

## Ethics Statement

Study approval was obtained from the Agence Française de Sécurité Sanitaire (Afssaps Ref B805-1860) and from the Comité de Protection des Personnes SUD Méditerranée I. The study was supervised by the Institut National de la Santé et de la Recherche Médicale (INSERM, protocol granted in 2008 under Ref ID RCB 2008-A00604-51, C07-17).

## Author Contributions

TL, SBa, SBu, HVC, VM, and YB were responsible for patient care, the selection of patients recruited in this study, the generation of clinical data, and the writing of the clinical sections of the manuscript. LL, DT, CL, and CD performed the flow cytometric analysis and the *in vitro* serum alloreactivity assay using NK-CHAT. CP was responsible for anti-HLA antibody characterization and the collection of HLA-typing and cross-match data and helped with the data interpretation and writing of the manuscript. AL contributed to the methodological design of the study and performed the statistical analyses. CB generated the data of the lymphocyte cell subset (% and cell counts) in KTR and healthy donors. LD was responsible for the anatomopathological characterization of biopsies and ABMR diagnosis. SM was responsible for the quality control of the regulatory aspects of the study, the coordination of ethical committee agreement, and the selection of healthy donors. FDG was responsible of the data generated in the hematology unit and the revision of the manuscript. PP was responsible for the design and coordination of the study, the quality control, analysis and interpretation of the data, and the writing of the manuscript.

## Conflict of Interest Statement

The authors declare that the research was conducted in the absence of any commercial or financial relationships that could be construed as a potential conflict of interest.
