# Supplementary material for: Antibody-Dependent NK Cell Activation Is Associated with Late Kidney Allograft Dysfunction and the Complement-Independent Alloreactive Potential of Donor-Specific Antibodies
Source: Front Immunol. 2016 Aug 11;7:288. doi: 10.3389/fimmu.2016.00288 (PMC4980873; doi:10.3389/fimmu.2016.00288)

Supplemental Figure S1

Test of the specific NK-CHAT reactivity of anti-HLA-A2 DSAs toward two distinct HLA-2 EBV-B cell lines or untransformed B cell targets

(A) Comparative analysis of rituximab and anti-HLA-A2 DSA$^+$ sera reactivity toward B cell targets. The NK-CHAT assay of anti-HLA-A2 DSA$^+$ serum obtained from a KTR patient prior to the initiation of ABMR therapy was performed using two distinct HLA-A2 EBV-transformed B cell lines. The NK-CHAT of DSA reactivity toward B cell targets expressing CD20 and HLA-A2 antigens was evaluated by the flow cytometric analysis of the serum-induced modulation of CD16 and CD107a/Lamp1 expression in NK cells exposed to target cells in the presence of either control DSA$^-$ serum (CTL Serum), CTL serum + 10 µg/mL rituximab, DSA$^+$ ABMR serum containing anti-HLA-A2 Abs, DSA$^+$ serum containing anti-HLA-A2 DSA or platelets with previously absorbed HLA-A2.

(B) Untransformed human leukocyte HLA-A2 cell targets were prepared using PBMCs freshly isolated from a healthy control blood donor following the depletion of NK cells.

(C) FCMX of anti-HLA-A2 and anti-HLA-A3 was tested on NK cell-depleted HLA-A2 PBMC targets. The specific binding of ABMR DSA$^+$ alloserum to NK cell-depleted HLA-A2 PBMC targets was analyzed by flow cytometry using an anti-human Fc PE secondary antibody (right panel).

(D) CD16 (MFI, upper panel) and CD107a/Lamp1 (%, lower panel) expression in NK cells was analyzed after incubation with targets including DSA$^+$ alloserum containing DSA with matched or unmatched specificities toward HLA-A antigens expressed on target PBMCs. Platelet absorption of serum HLA specificities partially reverted the alloserum-induced modification of CD16 and CD107a/Lamp1 expression in NK cells resulting from cognate HLA-class I antigen recognition by target allogeneic PBMCs.

Supplemental Figure S1

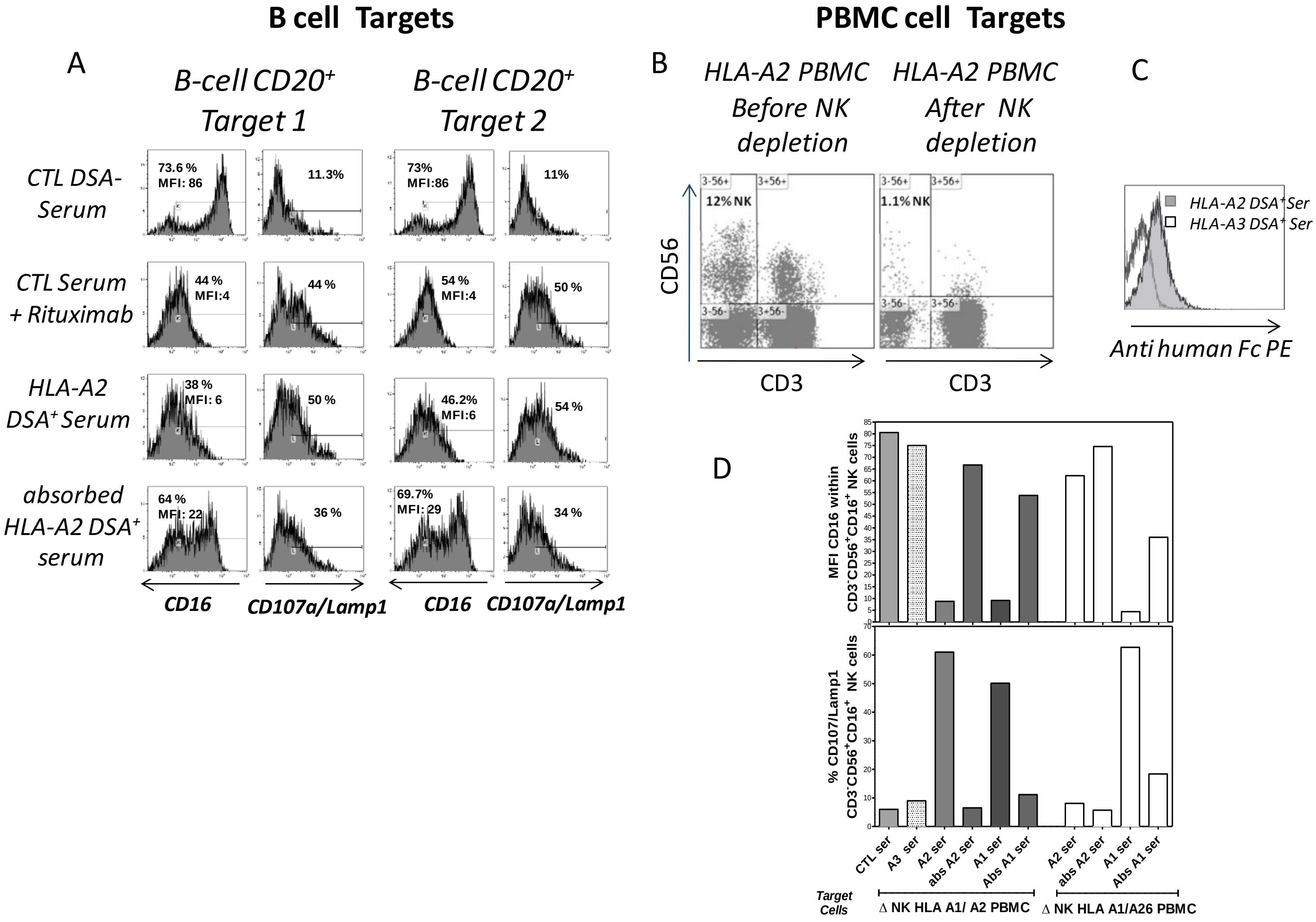

Supplement: Supplementary file 1 [file image_1.pdf]
