# Supplementary material for: Antibody-Dependent NK Cell Activation Is Associated with Late Kidney Allograft Dysfunction and the Complement-Independent Alloreactive Potential of Donor-Specific Antibodies
Source: Front Immunol. 2016 Aug 11;7:288. doi: 10.3389/fimmu.2016.00288 (PMC4980873; doi:10.3389/fimmu.2016.00288)

Supplemental Figure S2

The NK-CHAT scoring of DSA activity toward allogeneic cells isolated from donor spleen tissue

(A) Target cells were selected as splenic cells expressing HLA-A2 (antigenic targets of circulating DSA) or spleen cells that were not expressing HLA-A2. The target cell preparations were depleted from endogenous spleen NK cells prior to exposure of the serum. (B) NK cell expression of CD16 was evaluated after exposure of HLA-A2^{+} and HLA-A2^{-} spleen cells to CTL DSA^{-} or DSA^{+} alloserum containing anti-HLA-A2 antibodies.

(C) Illustration of flow cytometric analysis of CD16 and CD107a/Lamp1 expression in response to CTL DSA^{-} serum and DSA^{+} HLA-DR11 serum samples obtained during follow-up from a patient before transplant (D0) and at the times of rejection diagnoses at 90 and 140 months after transplantation (M90 and M140, respectively). Allogeneic splenic cells were selected for their expression of HLA-DR11 alloantigens that were recognized by the patient's DSAs.

Supplemental Figure S2

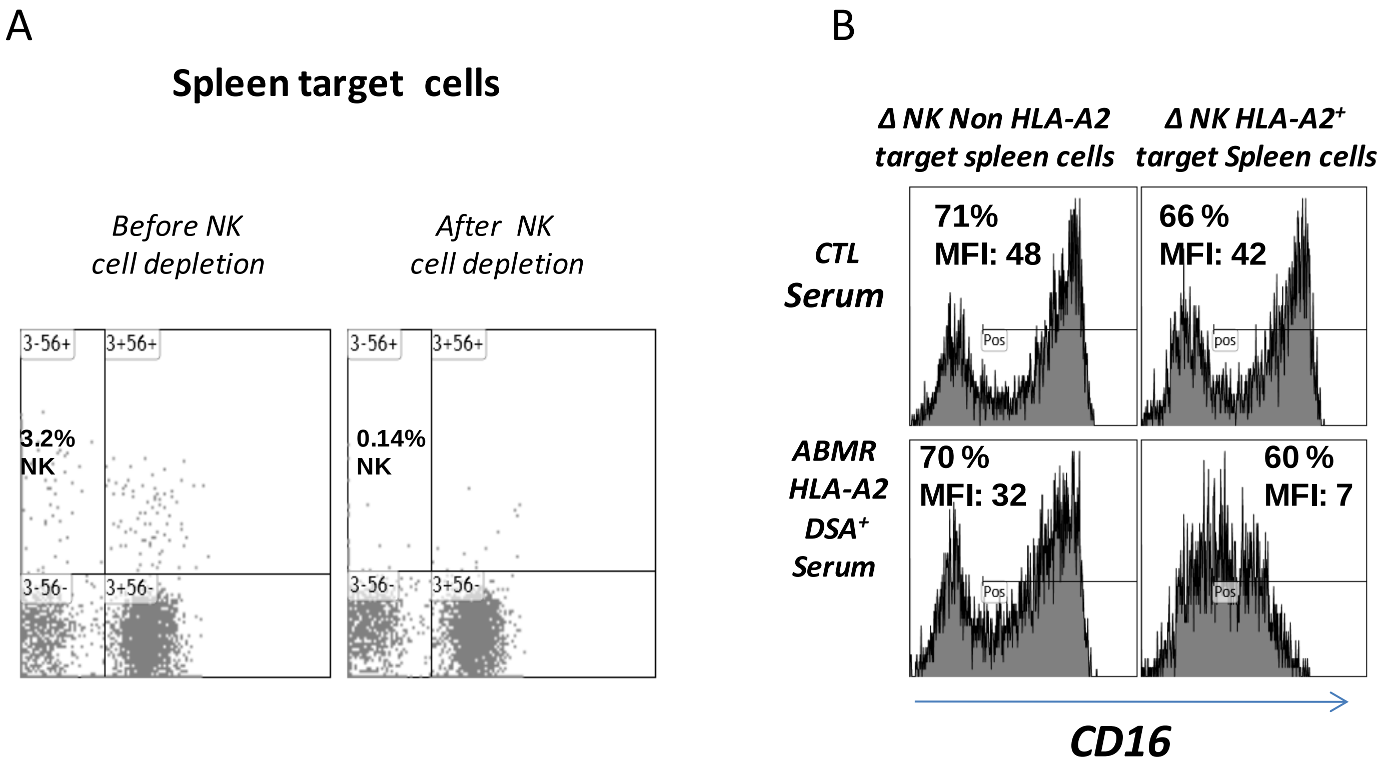

Supplement: Supplementary file 2 [file image_2.pdf]
