# Supplementary material for: Antibody-Dependent NK Cell Activation Is Associated with Late Kidney Allograft Dysfunction and the Complement-Independent Alloreactive Potential of Donor-Specific Antibodies
Source: Front Immunol. 2016 Aug 11;7:288. doi: 10.3389/fimmu.2016.00288 (PMC4980873; doi:10.3389/fimmu.2016.00288)

Supplemental Figure S3

ABMR sera with comparable anti-DQ7 MFIs show variable potential to activate FcR-dependent NK cell cytotoxicity toward allogeneic B cells

Serum-mediated CD16 NK cell engagement was measured after exposure of NK cells to B cells coated with various dilutions of DSA^+ serum obtained from two patients at the time of biopsy-proven ABMR (patient 06, serum anti-DQ7 MFI, 13,000; patient 08, DQ7 MFI, 15,000). CTL DSA^- human serum autologous with the PBMCs from the male donor used for isolation of the NK effector cells served as a reference for the CD16 MFI of NK cells (CD16 MFI: 95). The positive control for ADCC responsiveness was obtained by adding rituximab to CTL serum. The CD16DRI was calculated in response to 50% CTL serum, rituximab (CD16DRI: 15.8) or DSA contained in ABMR serum (serum S06 and serum S08). Despite a similar anti-DQ7 intensity, the CD16DRI values evaluated in response to the S08 ABMR serum using the same PBMC effector cells were lower than those observed in response to S06 (CD16 DRI: 3.2 versus 41). Variations in the CD16 DRI values observed in each patient were correlated with the ABMR serum dilution factor and the DSA MFI intensity in the diluted samples evaluated by Luminex.

Supplemental Figure S3

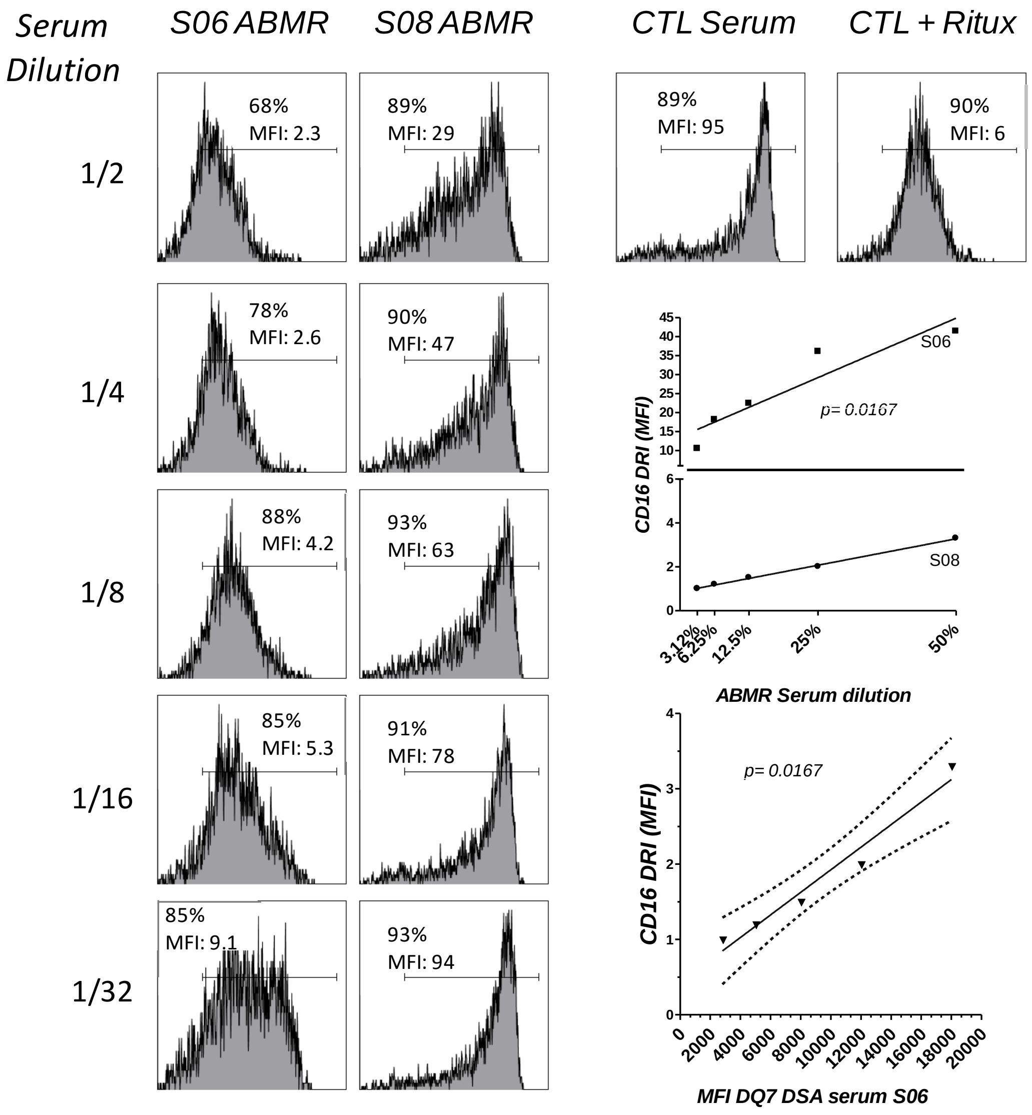

Supplement: Supplementary file 3 [file image_3.pdf]
